# MiRNA-424-5p Suppresses Proliferation, Migration, and Invasion of Clear Cell Renal Cell Carcinoma and Attenuates Expression of O-GlcNAc-Transferase

**DOI:** 10.3390/cancers13205160

**Published:** 2021-10-14

**Authors:** Thomas J. Kalantzakos, Travis B. Sullivan, Thales Gloria, David Canes, Alireza Moinzadeh, Kimberly M. Rieger-Christ

**Affiliations:** 1 Department of Translational Research, Lahey Hospital & Medical Center, Burlington, MA 01805, USA; thomas.kalantzakos@lahey.org (T.J.K.); travis.b.sullivan@lahey.org (T.B.S.); thalesgloria1@gmail.com (T.G.); 2 Department of Urology, Lahey Hospital & Medical Center, Burlington, MA 01805, USA; david.canes@lahey.org (D.C.); alireza.moinzadeh@lahey.org (A.M.)

**Keywords:** biomarkers, clear cell renal cell cancer (ccRCC), microRNA, O-GlcNAc-transferase (OGT), proliferation, migration, invasion

## Abstract

**Simple Summary:**

The identification of biomarkers that predict the metastatic potential of tumors is a current area of interest in cancer research. A previous study from our laboratory identified numerous microRNA (miRNA) biomarkers that are differentially expressed in pathologic stage I clear cell renal cell carcinoma (ccRCC) tumors that progress to metastatic disease. This study investigated the role of aberrant expression of one of these miRNA, miR-424-5p, and one of its associated protein targets, O-GlcNAc-transferase (OGT). We examined the influence of miR-424-5p and OGT expression on the proliferation, migration, and invasion of ccRCC cells, and confirmed the direct interaction between miR-424-5p and OGT. These findings suggest that the decrease in miR-424-5p expression observed in these small renal masses leads to an increase in OGT, which facilitates metastasis.

**Abstract:**

MicroRNAs (miRNAs) are non-coding post-transcriptional regulators of gene expression that are dysregulated in clear cell renal cell carcinoma (ccRCC) and play an important role in tumor progression. Our prior work identified a subset of miRNAs in pT1 ccRCC tumors, including miR-424-5p, that are associated with an aggressive phenotype. We investigate the impact of this dysregulated miRNA and its protein target O-GlcNAc-transferase (OGT) to better understand the mechanisms behind aggressive stage I ccRCC. The ccRCC cell lines 786-O and Caki-1 were used to assess the impact of miR-424-5p and OGT. Cells were transfected with pre-miR-424-5p, a lentiviral anti-OGT shRNA, or were treated with the demethylating agent 5-Aza-2′-deoxycytidine. Cell proliferation was measured via MT cell viability assay. Cell migration and invasion were analyzed using Transwell assays. The expression of miR-424-5p was determined through qRT-PCR, while OGT protein expression was evaluated through Western blotting. The interaction between miR-424-5p and OGT was confirmed via luciferase reporter assay. The transfection of ccRCC cells with pre-miR-424-5p or anti-OGT shRNA significantly inhibited cell proliferation, migration, and OGT expression, while miR-424-5p also attenuated cell invasion. Addition of the demethylating agent significantly reduced cell proliferation, migration, invasion, and OGT expression, while significantly increasing the expression of miR-424-5p. Altogether, these findings suggest that epigenetic downregulation of miR-424-5p, which in turn augments OGT expression, contributes to the creation of aggressive forms of stage I ccRCC.

## 1. Introduction

Renal cell carcinoma (RCC), arising from the renal epithelium, is a heterogeneous group of cancer variants accounting for over 90% of renal cancers and is among the ten most common cancers worldwide [1]. RCC subtypes can be further subdivided based on genetic, biological, or behavioral characteristics, with clear cell renal cell carcinoma (ccRCC) being the most common [2]. In addition to accounting for 75–80% of RCC incidence [2], ccRCC is overrepresented in metastatic RCC, accounting for greater than 90% of patients with advanced RCC [3]. Recent studies have focused on identifying new prognostic indicators of ccRCC progression in an effort to increase the personalization of treatment algorithms. Traditionally, the pathologic stage is the principal indicator of ccRCC progression [4], but with the rise of active surveillance of small tumors in today’s clinical landscape [5], new interests exist in methods to distinguish the metastatic potential of tumors of a similar pathologic stage. Studies have focused on a wide array of biomarkers to distinguish tumors that are more likely to progress to metastatic disease, such as transcription factors [6], long non-coding RNAs [7], and micro-RNAs (miRNA) [8]. One area of particular interest for identifying novel prognostic factors is within pathologic stage I (pT1, N0, Mx) ccRCC. While these tumors routinely yield favorable outcomes for the patient, cited in the 81–92% range [9], a small subset of these individuals will progress to metastatic disease.

A previous study from our laboratory further investigated this phenomenon by identifying miRNAs as potential biomarkers in identifying pT1 tumors that would progress to metastatic disease [9]. miRNAs are small, non-coding RNA molecules of about 20 nucleotides in length that post-transcriptionally bind to the 3′UTR of mRNA transcripts to suppress gene expression. The dysregulation of miRNAs contributes to cancer pathogenesis through their regulation of a wide range of mRNA transcripts [8]. As a result, miRNA dysregulation can exert a wide range of phenotypic effects depending on its protein targets, influencing key cellular processes, such as growth, development, differentiation, apoptosis, and metastasis [10]. miRNA expression profiles have also been demonstrated to be capable of identifying ccRCC patients with a higher risk of relapse following nephrectomy [11]. In ccRCC patients with pT1 ccRCC tumors, we identified miR-424-5p, among others, as downregulated in those that progressed to metastatic disease [9].

Previous studies are conflicting regarding the role of miR-424-5p in both ccRCC and a wide variety of other cancers. In kidney cell culture, expression of miR-424-5p was found to be reduced in ccRCC cells, compared to normal kidney cells, with overexpression of miR-424-5p resulting in inhibition of proliferation and induction of apoptosis [12]. Similarly, miR-424-5p has been demonstrated to play a tumor suppressor role in a wide range of other cancers [13,14,15,16,17,18], exerting its phenotypic impact on the cell by inhibiting proliferation, migration, and invasion while inducing apoptosis. Reduced expression of miR-424-5p has been connected to an increase in methylation of the miR-424 promoter CpG island in several cancer types [19,20,21,22]. Conversely, high expression of miR-424-5p has also been associated with ccRCC of high stage, grade, and progression [8], and miR-424-5p has been demonstrated as an oncogene in other cancers [23,24].

The influence of an individual miRNA on phenotype at the cellular level is through the specific pathways and protein targets through which they interact. One specific miRNA can interact with the mRNA transcripts of hundreds of different protein targets, while one protein target can be acted on by numerous miRNA [25]. The role an individual miRNA plays in tumorigenesis can be investigated by identifying the major protein targets and cellular pathways they impact. Algorithmic methods can be used to elucidate these targets, based on the target sequence and predicted base pairing stability [26]. Once potential protein targets are identified, experimental methods are needed to verify these interactions [25]. We used this method to elucidate the role of miR-424-5p in stage I ccRCC.

O-GlcNAc-transferase (OGT) is one protein target that is predicted to interact with miR-424-5p [27,28,29,30,31]. In addition, a study in mammary epithelial cell culture experimentally verified the interaction between miR-424-5p and OGT, identifying the former as a tumor suppressor [32]. OGT is an enzyme that post-transcriptionally modifies a target protein through the addition of an O-Linked β-N-acetylglucosamine (O-GlcNAc) sugar [33]. Imbalanced levels of OGT and O-GlcNAcylation is a hallmark of various cancers, and these imbalances play a role in promoting metastatic disease [33]. In ccRCC, OGT dysregulation has been demonstrated through immunohistochemistry [34]. In addition, patients with pT1 ccRCC tumors exhibiting high OGT expression tend to have worse outcomes than patients with low expression [35]. Experimentally, Wang et al. linked increased OGT expression in ccRCC with increased cell proliferation [34]. They also demonstrated the interaction between OGT and EGFR [34], a key protein involved in the epithelial–mesenchymal transition (EMT) cancer pathway. EMT confers increased migratory and invasive properties, in addition to chemoresistance, resulting in tumor progression and metastasis [36].

The aim of this study was to investigate the potential roles of miR-424-5p and OGT in cell proliferation, migration, and invasion and to further elucidate their potential interaction in ccRCC cells. We also aimed to investigate the potential impact of DNA methylation on the expression level of miR-424-5p and OGT in ccRCC. We investigated the link between this miRNA and protein to clarify the role of miR-424-5p in ccRCC cells in addition to improving our understanding of OGT’s role in metastasis.

## 2. Materials and Methods

### 2.1. Cell Culture

The human ccRCC cell lines 786-O (ATCC^®^ CRL-1932™) and Caki-1 (ATCC^®^ HTB-46™) (American Type Culture Collection, Manassas, VA, USA) were cultured under standard conditions (37°C, 5% CO_2_). 786-O, a VHL mutant cell line with altered HIF and VEGF pathways derived from a primary epithelial clear cell adenocarcinoma [37], was maintained in RPMI 1640 (ATCC). Caki-1, a VHL wild type cell line that is derived from a metastatic site on the skin and is characterized by high VEGF production [37], was grown in McCoy’s 5A media (ATCC). All media were supplemented with 10% fetal bovine serum, penicillin/streptomycin, and L-glutamine.

### 2.2. Prediction of miRNA Targets

A target search for predicted protein targets was conducted, using a combination of TargetScan (http://www.targetscan.org/, accessed on 6 April 2020) [27], micro-T CDS (http://diana.imis.athena-innovation.gr/, accessed on 6 April 2020) [28,29], RNAhybrid (https://bibiserv.cebitec.uni-bielefeld.de/rnahybrid, accessed on 6 April 2020) [30], and PicTar (https://pictar.mdc-berlin.de/, accessed on 6 April 2020) [31]. We chose a potential target based on predicted interactions on all search engines for the miR-15 family, which includes miR-424-5p [38].

### 2.3. Cell Transfection of Pre-miR Constructs

Cells were seeded into CELLSTAR six-well dishes (Greiner, Kremsmunster, Austria) at a density of 2 × 10^4^ cells/mL for both cell lines. Cells were transfected with 2 μM pre-miR-424-5p (Cat. #PM17100, Ambion, Austin, TX, USA), or 2 μM pre-miR-Precursor Negative Control #1 (Cat. #AM17110, Ambion, Austin, TX, USA) using siPORT NeoFX transfection reagent (Invitrogen, Carlsbad, CA, USA) according to the manufacturer’s protocol. RNA from a well of transfected cells was harvested using the Qiagen miRNeasy mini kit (Qiagen, Hilden, Germany). The RNA quantity and purity (OD 260/280 ratio) were accessed using the Epoch spectrophotometer (BioTek, Winooski, VT, USA). The expression of miR-424-5p compared to the control, RNU43, was analyzed using Taqman miRNA qRT-PCR assays according to the manufacturer’s instructions (assay ID: 000604 and 001095, Applied Biosystems, Foster City, CA, USA), using 5 ng of RNA per sample in a 20 μL reaction volume for RT. The resulting cDNA was quantified in a 20 μL reaction volume, assayed in triplicate, on the C1000 Touch Thermal Cycler using the CFX Maestro software v4.0.2325.0418 (Bio-Rad, Hercules, CA, USA). The normalization of miR-424-5p expression to RNU43 was conducted, using the comparative C(T) method [39]

### 2.4. Cell Proliferation Assay

Cells were seeded at a density of 2 × 10^4^ cells/mL and transfected with either pre-miR-424-5p or pre-miR-Precursor Negative Control #1. At 48 h after transfection, RealTime-Glo^™^ MT Cell Viability Assay (Promega, Madison, WI, USA) reagents were added, at a 1:2000 dilution. Measurements were taken one hour after addition of the assay reagents in the GloMax^®^ 20/20 Luminometer (Promega). This process was repeated 72 h after cell transfection. Each experiment was set up in duplicate.

### 2.5. Cell Migration and Invasion Assays

In vitro migration and invasion assays were carried out 48 h after transfection using modified Boyden chambers consisting of Transwell (8 μm pores; Corning Costar Corp., Cambridge, MA, USA) membrane filter inserts in 24-well tissue culture plates. For invasion assays, the upper surfaces of the membranes were coated with Matrigel (Becton-Dickinson, Franklin Lakes, NJ, USA) diluted 1:80 and placed into 24-well plates containing 10% RPMI for 786-O and 20% McCoy’s for Caki-1 cells. No Matrigel was used for migration assays where the chemoattractant used was fibronectin (10 μg/mL). In migration assays, cells (1 × 10^5^ cells/mL) were added to each Transwell chamber, and allowed to migrate toward the underside of the membrane for 20 h at 37 °C. For invasion assays, 786-O (1 × 10^5^ cells/mL) or Caki-1 (5 × 10^5^ cells/mL) were added to each Transwell chamber and allowed to invade toward the underside of the membrane for 30 h at 37 °C. Cells that passed through the membrane were fixed in 10% *w*/*v* neutral-buffered formalin (Simport, Beloeil, QC, Canada), stained with DAPI (Invitrogen) (1:500 dilution in PBS, 1% Triton X-100), and counted using a microscope (Evos; Advanced Microscopy Group, Bothwell, WA, USA). Three unique image frames were used for each transwell, which were run in triplicate for each experiment.

### 2.6. Western Blot Analysis

At 48 h after transfection, dishes displaying 70–80% confluency were used to prepare cell lysates. Cells were lysed by manually scraping dishes in 100 μL/well of boiled 1 × SDS-Laemmli (250 mM Tris-HCl, 4% SDS, 10% glycerol, 0.003% bromophenol blue), followed with shearing by a 24-gauge needle. BCA assay (Pierce, Waltham, MA, USA) was used to determine the concentration of each protein sample. Paired NC and miR-424-5p lysates were standardized for volume and total protein concentration, along with 2 μL/100 μL βME. Each lysate was boiled for 5 min following the addition of βME, and loaded into a unique lane in a 4–15% gradient polyacrylamide gel (Mini-PROTEAN^®^ TGX™, Bio-Rad). The iBlot^™^ 2 gel transfer system (Invitrogen) was used to transfer proteins onto nitrocellulose, which was then blocked in 10% milk in TBS with 0.05% Tween (TBST) for 3 h. Membranes were placed on OGT primary antibody (ab177941, Abcam, Cambridge, UK) at a concentration of 1:1000 overnight at 4 °C. Following a TBST wash, the blots were placed on rabbit specific secondary antibody (NA934V, Amersham Biosciences, Amersham, UK) at a concentration of 1:1000 for 1 h at room temperature. The blots were developed using an ECL kit (Pierce), and densitometry was performed using the ImageJ software (National Institute of Health, Bethesda, MD, USA) as previously described [40]. OGT values were normalized using GAPDH primary antibody (2118, Cell Signaling Technology, Danvers, MA, USA) at a concentration of 1:2000.

### 2.7. 5′ Aza Treatment of Cells

Cells were seeded into CELLSTAR six-well dishes at a density of 2 × 10^4^ cells/mL. 24 h later, cells were treated with 10 μM 5-Aza-2′-deoxycytidine (5′Aza) (Sigma Aldrich, St. Louis, MO, USA), or DMSO as negative control, for 48 h, replacing the media after 24 h. Following treatment with 5′Aza, cell RNA and protein was isolated as previously described. qRT-PCR was conducted to analyze expression of miR-424-5p at each condition normalized against RNU43. Western blotting assessed the expression level of OGT at each condition normalized against GAPDH. qRT-PCR and Western blotting were repeated for a minimum of 4 independent experiments per condition. Treated cells were assayed for proliferation, migration, and invasion following the same protocol as for the pre-miR transfections.

### 2.8. Lentiviral Infection of OGT shRNA

Lentiviral shRNA constructs for human OGT were obtained from Origene (Rockville, MD, USA). Stable cell lines were established from each OGT shRNA construct as well as negative control constructs according to the manufacturer’s protocol. Unique shRNA sequences were used for each cell line (version TL302811VB for 786-O and version TL302811VD for Caki-1; see Figure S2 for details). Expression levels of OGT were determined by Western blot analysis. OGT expression levels were normalized against GAPDH expression levels. Following confirmation of OGT knockdown in each line, stable cell lines were assayed for proliferation and migration, following the same protocol as for the pre-miR transfections.

### 2.9. Luciferase Assay

Vectors were prepared containing the full-length human OGT 3′UTR linked to the Firefly luciferase gene in addition to the Renilla luciferase gene for normalization (Genecopoeia, Rockville, MD, USA). The OGT 3′UTR contained the theoretical interaction sites between miR-424-5p and OGT. Additional vectors were created to assess each potential binding site through site directed mutagenesis. The 786-O cells were seeded at a density of 5 × 10^4^ cells/mL into CELLSTAR 24-well dishes. After 24 h, the media was replaced with Opti-MEM™ Reduced Serum Medium (31985062, Invitrogen). Cells were transfected with the vector (0.2 μg) along with either pre-miR-424-5p or pre-miR-Precursor Negative Control #1 (30 nM), delivered with Endofectin (Genecopoeia, Rockville, MD, USA). 24 h after transfection, cells were lysed following the manufacturer’s protocol, and luminescence was measured using the Luc-Pair^™^ Duo-Luciferase Assay kit (Genecopoeia, Rockville, MD, USA). Firefly luminescence was normalized relative to Renilla luminescence.

### 2.10. Statistical Analysis

A two-tailed Welch’s *t*-test was conducted (SPSS v26) to determine whether a statistically significant difference in traits exists for cells undergoing treatment, compared to negative control. Corresponding plots depict the mean relative response rate for treated cells relative to negative control. Error bars represent the standard error of the mean. A *p*-value < 0.05 was considered statistically significant. All assays were performed with at least four separate experiments for each cell line.

## 3. Results

### 3.1. Overexpression of miR-424-5p Inhibits In Vitro Proliferation, Migration, and Invasion of 786-O and Caki-1 Cells

**Figure 1:**
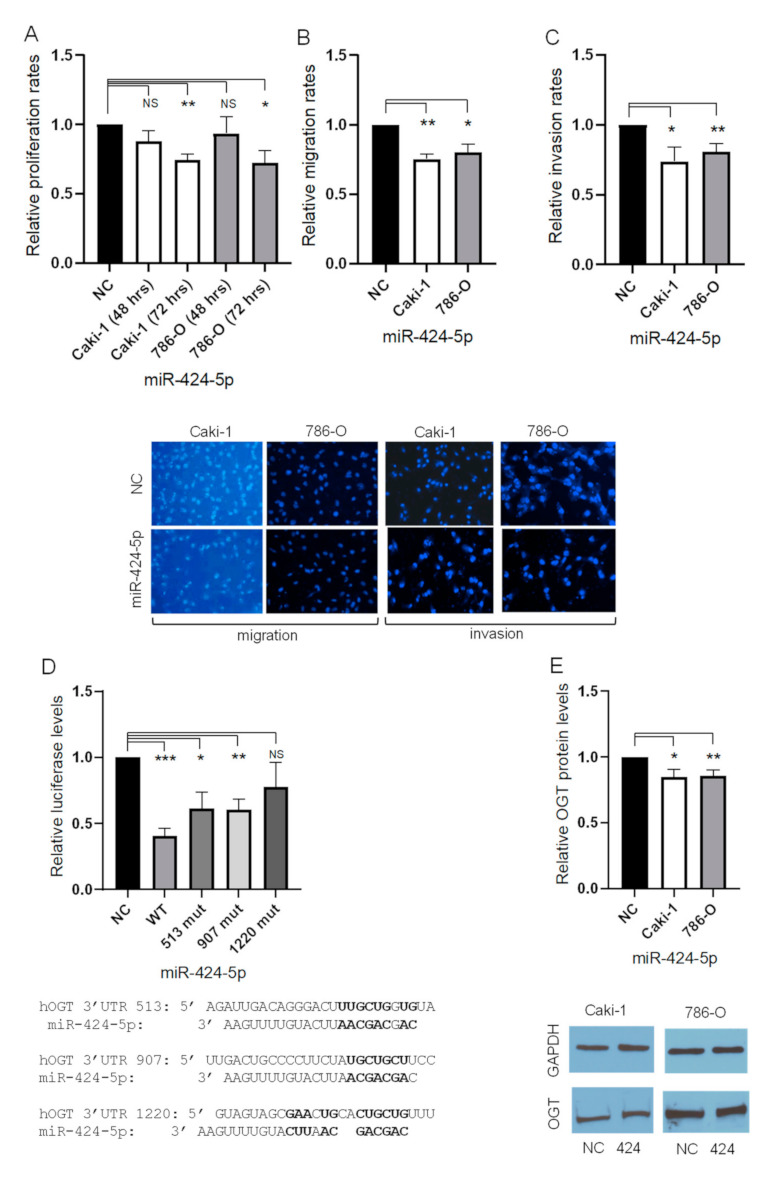
The effect of miR-424-5p on the ccRCC phenotype and expression of OGT. (**A**) The proliferation rates of cell lines transfected with pre-miR-424-5p after 48 h and 72 h, relative to negative control transfectants. (**B**) The migration rates of cell lines transfected with pre-miR-424-5p after 20 h, relative to negative control transfectants (representative images below, 20× magnification). (**C**) The invasion rates of cell lines transfected with pre-miR-424-5p after 30 h, relative to negative control transfectants (representative images below, 20× magnification). (**D**) The luciferase activity based on the WT 3′UTR as well as three mutated sites of the 786-O cell line transfected with pre-miR-424-5p, relative to negative control transfectant (predicted sites detailed below). (**E**) The OGT protein expression of cell lines transfected with pre-miR-424-5p after 48 h, relative to negative control transfectants (representative images below) (Figures S3–S6). * *p* < 0.05, ** *p* < 0.01, *** *p* < 0.001, and NS = not significant.

Restoration of miR-424-5p expression in 786-O and Caki-1 cell lines was performed using siPORT NeoFX transfection reagent with pre-miR-424-5p and a scrambled sequence as the control. Forty-eight hours post-transfection, cells were tested in a standard proliferation assay. While the proliferation of 786-O and Caki-1 cells was not significantly altered after 48 h, transfection with miR-424-5p significantly reduced proliferation after 72 h when compared to the negative control (Figure 1A). To further investigate the role of miR-424-5p in ccRCC, we performed cell migration and invasion assays. miR-424-5p transfection of 786-O and Caki-1 cells significantly reduced cell migration compared to transfection with a negative control miRNA construct (Figure 1B). Similarly, overexpression of miR-424-5p significantly reduced the invasive capacity of 786-O and Caki-1 cells compared to negative control (Figure 1C).

### 3.2. miR-424-5p Directly Regulates OGT by Targeting its 3′UTR

All prediction algorithms utilized in this study identified OGT as a target of the miR-15 family [27,28,29,30,31]. Although several potential 3′UTR binding sites were identified, we investigated the three sites that were identified by two or more of the algorithms. These sites are referred to as 513, 907, and 1220 based on their location in the 3′UTR of OGT (see Figure 1D and Figure S1. A dual luciferase reporter assay was performed in an effort to determine whether miR-424-5p directly targets the 3′UTR of OGT in ccRCC line 786-O (Figure 1D). Unique plasmids were constructed to assess the activity of each predicted binding site. Renilla/Firefly luminescence ratio was calculated to compare the luciferase assay between groups. Co-transfection with miR-424-5p and the wild-type 3′-UTR of OGT showed significantly lower luciferase activity, compared to the negative control group. In contrast, the luciferase activity of the cells transfected with miR-424-5p and the 1220 mutant was not significantly different than the control. When miR-424-5p was co-transfected with either the 513 or the 907 mutant, the luciferase activity was significantly lower than the negative control but higher than the wild-type. Western blot analysis was conducted to validate the influence of miR-424-5p on OGT expression in ccRCC cells as seen with our luciferase reporter assay. As shown in Figure 1E, transfection of 786-O and Caki-1 cells with miR-424-5p significantly reduced OGT protein expression compared to cells transfected with the negative control pre-miRNA construct.

### 3.3. Treatment with 5′Aza Reduces Proliferation, Migration, and Invasion with Altered Expression of miR-424-5p and OGT in ccRCC Cells

**Figure 2:**
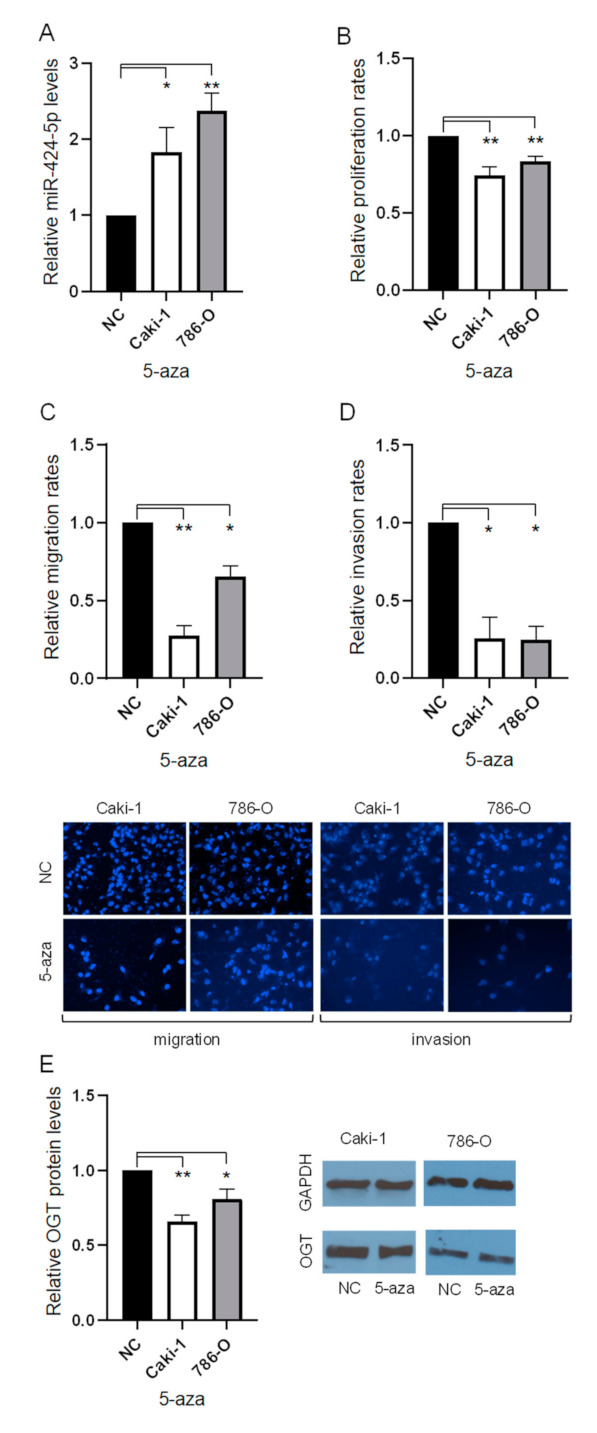
The effects of 10 μM 5-Aza-2′-deoxycytidine on the ccRCC phenotype and expression of OGT. (**A**) The expression of miR-424-5p in cell lines treated with 5′aza after 48 h, relative to negative control treatment. (**B**) The proliferation rates of cell lines treated with 5′aza after 48 h, relative to negative control treatment. (**C**) The migration rates of cell lines treated with 5′aza, relative to negative control treatment (representative images below, 20× magnification). (**D**) The invasion rates of cell lines treated with 5′aza, relative to negative control treatment (representative images below, 20× magnification). (**E**) The OGT protein expression of cell lines treated with 5′aza after 48 h, relative to negative control treatment (representative images aside) (Figures S7–S10). * *p* < 0.05 and ** *p* < 0.01.

Methylation of the miR-424-5p CpG island promoter has been identified as a cause of reduced expression of miR-424-5p in numerous other cancer types [19,20,21,22]. However, the impact of methylation on expression of miR-424-5p in ccRCC has not yet been established. The 786-O and Caki-1 cells were treated with 10 μM of the demethylation drug 5′Aza to establish its impact on cell behavior. ccRCC cells treated with 5′Aza expressed miR-424-5p at significantly higher rates than cells treated with negative control (Figure 2A). The impact of 5′Aza treatment on cell proliferation, migration, and invasion was also evaluated in both 786-O and Caki-1 cells. The 5′Aza addition significantly attenuated proliferation, migration, and invasion in 786-O and Caki-1 cells (Figure 2B–D). In addition, 786-O and Caki-1 cells treated with 5′Aza showed significantly reduced expression of OGT, compared to negative control (Figure 2E).

### 3.4. OGT Lentiviral Knockdown Suppresses ccRCC Cell Proliferation and Migration

**Figure 3:**
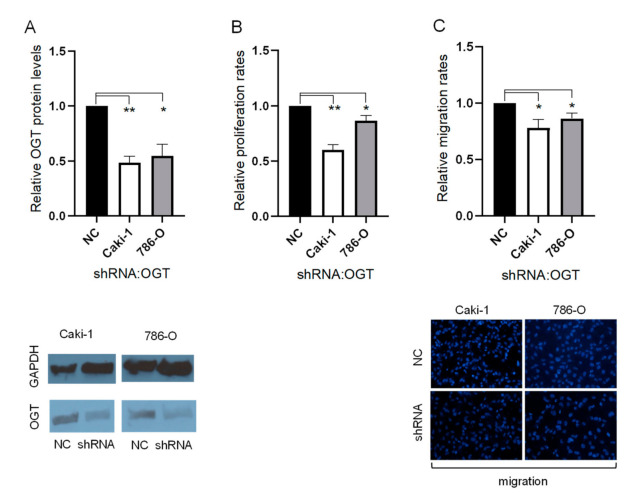
The effects of OGT knockdown on the ccRCC phenotype. (**A**) The OGT protein expression of cell lines treated with OGT shRNA, relative to negative control treatment (representative images below). (**B**) The proliferation rates of cell lines treated with OGT shRNA, relative to negative control treatment. (**C**) The migration rates of cell lines treated with OGT shRNA, relative to negative control treatment (representative images below, 20× magnification) (Figures S11–S12). * *p* < 0.05 and ** *p* < 0.01.

OGT was identified as a direct target of miR-424-5p. Therefore, we hypothesized that miR-424-5p suppresses cell proliferation and migration in ccRCC via inhibition of OGT expression. To confirm this hypothesis, endogenous OGT expression was knocked down in 786-O and Caki-1 cells, using OGT shRNA lentiviral knockdown. Stable OGT knockdown was successfully completed in both 786-O and Caki-1 cells (Figure 3A). Confirmation that OGT knockdown significantly diminished cell proliferation and cell migration in ccRCC cells is shown in Figure 3B,C.

## 4. Discussion

The prognostic capability of miRNAs has been investigated in numerous studies, with their impact driven through altered protein expression of tumor suppressors and oncogenes. Our previous study identified miR-424-5p, among others, as downregulated in small stage I ccRCC tumors that would later progress to metastatic disease [9]. This study investigated the impact and interaction of miR-424-5p and OGT on the proliferation, migration, and invasion of ccRCC cells. While an individual miRNA can act on many potential protein targets, we have gained insight into the relationship between miR-424-5p and OGT, an oncogene responsible for post-transcriptionally modifying other proteins through the addition of an O-GlcNAc sugar [33].

The current study demonstrated a tumor suppressor role for miR-424-5p on the cancer phenotype of ccRCC cells. Cells transfected with pre-miR-424-5p exhibited a significantly reduced proliferation rate, compared to cells transfected with a negative control in Caki-1 and 786-O ccRCC cell lines. Similarly, cell migration and invasion were both attenuated through transfection with pre-miR-424-5p, compared to the negative control in both ccRCC lines. Treatment of ccRCC cells with 10 uM 5′Aza significantly increased the expression of miR-424-5p and decreased the expression of OGT. As seen with direct transfection of miR-424-5p, treatment of ccRCC cells with 5′Aza resulted in significantly reduced cellular proliferation, migration, and invasion. Lentiviral shRNA knockdown of OGT in ccRCC cells produced a similar impact to transfection with miR-424-5p and the addition of 5′Aza. Lentiviral OGT knockdown significantly reduced cell proliferation and migration. Through Western blot and luciferase analysis, we confirm that miR-424-5p decreases OGT expression and binds to the 3′UTR. We hypothesize that the attenuation of miR-424-5p expression, along with the overexpression of OGT in ccRCC cells, is at least partially driven by increased methylation of the promoter of miR-424-5p. Immunohistochemical staining of ccRCC tissues demonstrated increased expression of OGT in ccRCC tumors, compared to adjacent normal tissue [34]. In addition, the TCGA dataset presented by the Human Protein Atlas indicates that OGT upregulation is associated with poorer patient outcomes in all stages of ccRCC as well as in stage I patients alone [35,41].

Studies on miR-424-5p in ccRCC, as well as in other cancers, have shown conflicting results regarding its role. In addition to our previous study in stage I ccRCC tumors, Chen et al. showed that miR-424-5p functions as a tumor suppressor and is downregulated in ccRCC, compared to normal kidney tissue, with elevated miR-424-5p expression inhibiting proliferation and inducing apoptosis [12]. In addition to ccRCC, the downregulation of miR-424-5p has been linked to increased tumorigenesis in a wide range of other cancers, including ovarian [13], nasopharyngeal [14], intrahepatic cholangiocarcinoma [15], endometrial [16], cervical [17], and basal-like breast cancer [18]. Conversely, Gowrishankar et al. identified miR-424-5p upregulation as being associated with ccRCC progression and poorer prognosis [8]. miR-424-5p was also identified as an oncogene in laryngeal squamous cell carcinoma [23] and colorectal cancer [24]. Our present study supports the tumor suppressor activity of miR-424-5p in ccRCC, suggesting that low expression of miR-424-5p in ccRCC contributes to tumor aggressiveness. Studies have also investigated the methylation status of miR-424-5p in a wide range of cancers. In glioma clinical specimens and cell lines, methylation of the CpG island promoter for miR-424-5p was directly correlated with high grades [19]. Similarly, in bladder cancer, tumor progression is regulated by DMNT dependent methylation of the CpG island promoter [20]. Reduced expression of miR-424-5p was also seen in ovarian cancer [21] and colorectal cancer [22] cells, stemming from increased methylation of the miR-424-5p promoter. Our present studies on the impact of 5′Aza on miR-424-5p expression support the idea that reduced miR-424-5p expression in aggressive stage I ccRCC stems from increased DNA methylation. While the methylation status of miR-424-5p and underpinning mechanisms behind it has not been explicitly determined in aggressive ccRCC, we hypothesize that low miR-424-5p expression originates from hypermethylation in the CpG island promoter. A limitation of this study is that we did not perform methylation analysis of the miR-424-5p promoter specifically, but we expect to include this in a future study investigating methylation in small renal masses.

While miR-424-5p likely targets the 3′UTR of many protein transcripts, we chose to focus on OGT, due to its predicted interaction with miR-424-5p and its correlation with patient prognosis in ccRCC [35]. OGT functions by mediating the addition of an O-GlcNAc sugar to a target protein [42], giving OGT a wide-reaching impact on cell behavior [43]. Wang et al. illustrated decreased cell proliferation when knocking down OGT in RCC cells [34], linking OGT to the EMT cancer pathway. Our present knockdown of OGT confirms the impact of OGT on the mesenchymal phenotype, as OGT knockdown significantly decreased cell migration in ccRCC cells. Another area that OGT has been implicated is epigenetics, specifically DNA methylation [43]. While OGT’s impact on methylation has not been extensively studied in ccRCC, OGT has been associated with proteins involved with methylation and demethylation pathways [44]. OGT catalyzes the addition of an O-GlcNAc sugar to the TET family of proteins, which actively mediate DNA demethylation at CpG island promoters [43,44]. In addition to demethylation pathways, OGT has also been implicated in DNA methylation through its association with TRIM28 [44]. Boulard et al. found that DNA methylation promotes the formation of OGT-TRIM28 complexes, which can amplify the inhibition of gene expression [45]. TRIM28 has also been identified for its repression of miRNA expression [46]. Taken together, further study is needed to understand the role of OGT on the function of the TET proteins and TRIM28 in ccRCC. This avenue of study could elucidate the mechanism behind the methylation-induced downregulation of microRNAs, such as miR-424-5p in ccRCC.

The link between these two factors was previously investigated in mammary epithelial cells, noting that miR-424-5p did indeed bind to the 3′UTR of OGT [32]. Vaiana et al. used a luciferase reporter assay to verify the binding of miR-424-5p to the 907 site. However, their site mutagenesis work only partially diminished the effect of miR-424 on OGT, and they therefore speculated the presence of other miR-424 binding sites [32]. We mutated three binding sites predicted for miR-424-5p, all of which showed less activity than the wild-type construct. Two of the three constructs (sites 513 and 907) produced significantly more interaction than negative control but less than WT, suggesting that miR-424-5p binds primarily to the remaining site (site 1220) but also partially to the others. One limitation of the present study is that we did not investigate site mutagenesis for all predicted sites simultaneously. There are also additional sites that were identified using other prediction algorithms, though we chose to look at a consensus of sites from four algorithms. As such, it is possible that there are additional sites where miR-424-5p binds to the OGT transcript that we did not investigate.

## 5. Conclusions

In a previous study, we observed the downregulation of miR-424-5p in aggressive stage I ccRCC tumors, and in this study, we demonstrate that this contributes to the upregulation of OGT in ccRCC cell lines. This downregulation is likely at least partly attributed to methylation of the miR-424-5p promoter. We demonstrated that miR-424-5p modulates proliferation, migration, invasion, and OGT expression in ccRCC cells. We also further describe the likely role of OGT in metastasis through the migratory ability it confers.

## Data Availability

The data presented in this study are available on request from the corresponding author.

## Supplementary Materials

The following are available online at https://www.mdpi.com/article/10.3390/cancers13205160/s1, Figure S1: OGT 3′UTR vector. (**A**) Vector map for the Genecopoeia vector containing the 3′UTR of OGT. (**B**) The sequence of the 3′UTR of OGT. The entire sequence of the OGT 3′UTR was inserted into the vector. Four separate vectors were utilized containing unique 3′ UTRs: wild type, mutation targeting region 513, mutation targeting region 907, and mutation targeting region 1220, Figure S2: shRNA OGT vector. (**A**) Map for the Origene Lenti Cloning Vector (pGFP-C-shLenti Vector) with shRNA against OGT. (**B**) The shRNA sequences utilized in this study, Figures S3–S12: Original western blots.
