# Supplementary material for: MiRNA-424-5p Suppresses Proliferation, Migration, and Invasion of Clear Cell Renal Cell Carcinoma and Attenuates Expression of O-GlcNAc-Transferase
Source: Cancers (Basel). 2021 Oct 14;13(20):5160. doi: 10.3390/cancers13205160 (PMC8533684; doi:10.3390/cancers13205160)
Supplement: Supplementary file 1 [file cancers-13-05160-s001.zip › cancers-1404558-supplementary.pdf]

MiRNA-424-5p Suppresses Proliferation, Migration, and Invasion of Clear Cell Renal Cell Carcinoma and Attenuates Expression of O-GlcNAc-Transferase

Thomas J. Kalantzakos, Travis B. Sullivan, Thales Gloria, David Canes, Alireza Moinzadeh and Kimberly M. Rieger-Christ

(A)

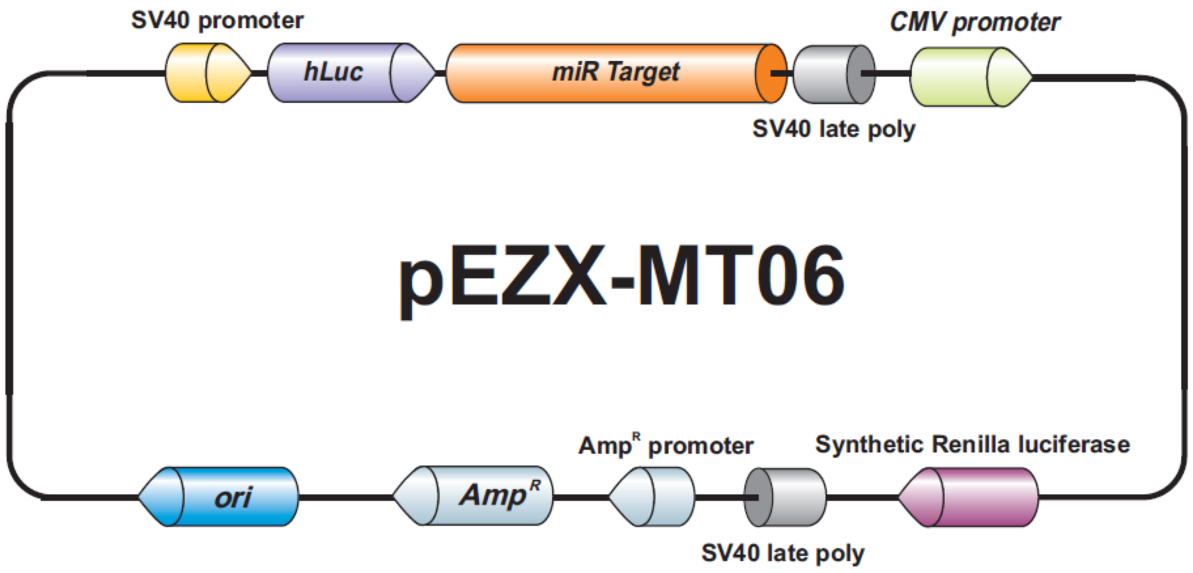

(B)

3’UTR OGT (NM_181673.3)

Wild type

ataaagactgcacaggagaattacccctatacctgagcctcaaccttctgggggaaaggg
aactagataacatacttcttacttgtctgtacagtaccttgttgcagatgggtgatatat
aatggtaatagaatagcacagccagacttgcttcctgcatggtagggagagacacaaaag
atgggaaactgcttttccacaaggaatctccgtagaattttgcggcgaccagatggtgca
taggtctggaaggtctgatctcccttggtcttccatgggatggttagtgtggaggggaga
tatagattgtccggccgctttgtgattccatggattgattcagtcttctggatttttttt
tctttatattttgggtactggagcttttaaaaatgtttggtttcaggtatttttattcat
gtgaagtgtatatgattctcttgagataaggttttaagctaaaatgttactccctgtttt
agtttctgaactctgacagattgacagggactttgctggtgtagtctttttataggtttt
ataaaccacttgagcctatatcagtcgttttagtgtctgacctaatatttggagctatca
gtgctttgttgatttagatgatgactcaagattttttctggtccatttcccatttccttt
tcttccctgacccccataccctcacccttaaaattctcctgtaactcaactaacaaaatc
aagcctgattcaaaacatcctagggtgttttaaacacaccatctggtgccaaatgaagat
ttttaggagtgattactaattatcaagggcacagttgtggtactgtcattgataataata
tagtttttttttttttcctaattttgacctgtttcaccagtgttttacccttgactgccc
cttctatgctgcttccaaaagtgatagtgtgtgtaagatttttaccttcctttctaaagt

tttttttttttttttttaagtgagtcctgttcttcctatttctttcagcagaaatgaaat
cccaggtaagtataagtattcaagtatttgatcagtaagtcacagttatctccagtgcat
taaataaccttcatcaagaaataggttataggtaaaatctctgaaggatcatctatgtat
tcaagtaattattttttagataataactgtcttctggacttggtcttgaagtctgtacag
attcagcctcagtagtagcgaactgcactgctgtttggtttggagtacaaattagactta
tagtcctcctggaacttgagttattaaaatcataggaataaaattatgggatctcaacaa
agggtcgagggtttgaggcttaaacaagccaacatatgaatatatgttttgtctcgctat
actgcacttacgctatccagttgcaggtaatttttgtctgctagtagtgttctagatta
tgtctttccaaagcgctgaggctgtgcacctattctgtagttgcagctgatgcctgaatg
tatcctagctgacaaattattgattaataagaacttgaatttctggaagattcttactgt
taaccaaattttgagcaaggagtctcaaaggtaattctgaaccagaattacatgttaatg
aacagtgtaccttttaacagtgtaaatcacggaatatccgtgaagggatttcttaattta
ttttttaccggttgattgaaatatcagttaaaggttgccagcatggttgcagataaactg
atgtttgaaattcgctgaaatacttaatgtggaataggataatatacttccaatgccctc
aaggctgtgaccttacagccattttacatagcacatcattcctcctatagggatgaactt
tttcctggcacgaaaagtagccgctctggttgaagctttgcttattgtaacaggctttta
tttccaggtaatatgtcttggaagacttaattctgattagagatatagatattactggaa
actaattgttttttttctattgtactctgctttatcaaagaagtaaaacatttaaatcgt
actacagaaattaagatgttgtcttgcgatccttaataaatgaatgatttccctttaa

mutation targeting region 513
ataaagactgcacaggagaattacccctatacctgagcctcaaccttctgggggaaaggg
aactagataacatacttcttacttgtctgtacagtaccttgttgcagatgggtgatatat
aatggtaatagaatagcacagccagacttgcttcctgcatggtagggagagacacaaaag
atgggaaactgcttttccacaaggaatctccgtagaattttgcggcgaccagatggtgca
taggtctggaaggtctgatctcccttggtcttccatgggatggttagtgtggaggggaga
tatagattgtccggccgctttgtgattccatggattgattcagtcttctggattttttttt
tctttatattttgggtactggagcttttaaaaatgtttggtttcaggtattttttattcat
gtgaagtgtatatgattctcttgagataaggttttaagctaaaatgttactccctgtttt
agtttctgaactctgagtgtttgtgtgcgtctaacgacgagtagtctttttataggtttt
ataaaccacttgagcctatatcagtcgttttagtgtctgacctaatatttggagctatca
gtgctttgttgatttagatgatgactcaagatttttttctggtccatttcccatttccttt
tcttccctgacccccataccctcacccttaaaattctcctgtaactcaactaacaaaatc
aagcctgattcaaaacatcctagggtgttttaaacacaccatctggtgccaaatgaagat
ttttaggagtgattactaattatcaagggcacagttgtggtactgtcattgataataata
tagtttttttttttttcctaattttgacctgtttcaccagtgttttacccttgactgccc
cttctatgctgcttccaaaagtgatagtgtgtgtaagattttttaccttcctttctaaagt
tttttttttttttttttaagtgagtcctgttcttcctatttctttcagcagaaatgaaat
cccaggtaagtataagtattcaagtatttgatcagtaagtcacagttatctccagtgcat
taaataaccttcatcaagaaataggttataggtaaaatctctgaaggatcatctatgtat
tcaagtaattattttttagataataactgtcttctggacttggtcttgaagtctgtacag
attcagcctcagtagtagcgaactgcactgctgtttggtttggagtacaaattagactta

tagtcctcctggaacttgagttattaaaatcataggaataaaattatgggatctcaacaa
agggtcgagggtttgaggcttaaacaagccaacatatgaatatatgttttgtctcgctat
actgcacttacgctatccagttgcaggtaattttttgtctgctagtagtgttctagatta
tgtctttccaaagcgctgaggctgtgcacctattctgtagttgcagctgatgcctgaatg
tatcctagctgacaaattattgattaataagaacttgaatttctggaagattcttactgt
taaccaaattttgagcaaggagtctcaaaggtaattctgaaccagaattacatgttaatg
aacagtgtaccttttaacagtgtaaatcacggaatatccgtgaagggatttcttaattta
ttttttaccggttgattgaaatatcagttaaaggttgccagcatggttgcagataaactg
atgtttgaaattcgctgaaatacttaatgtggaataggataatatacttccaatgccctc
aaggctgtgaccttacagccattttacatagcacatcattcctcctatagggatgaactt
tttcctggcacgaaaagtagccgctctggttgaagctttgcttattgtaacaggctttta
tttccaggtaatatgtcttggaagacttaattctgattagagatatagatattactggaa
actaattgttttttttctattgtactctgctttatcaaagaagtaaaacatttaaatcgt
actacagaaattaagatgttgtcttgcgatccttaataaatgaatgatttccctttaa

mutation targeting region 907
ataaagactgcacaggagaattacccctatacctgagcctcaaccttctgggggaaaggg
aactagataacatacttcttacttgtctgtacagtaccttgttgcagatgggtgatatat
aatggtaatagaatagcacagccagacttgcttcctgcatggtagggagagacacaaaag
atgggaaactgcttttccacaaggaatctccgtagaattttgcggcgaccagatggtgca
taggtctggaaggtctgatctcccttggtcttccatgggatggttagtgtggaggggaga
tatagattgtccggccgctttgtgattccatggattgattcagtcttctggatttttttt
tctttatattttgggtactggagcttttaaaaatgtttggtttcaggtatttttattcat
gtgaagtgtatatgattctcttgagataaggttttaagctaaaatgttactccctgtttt
agtttctgaactctgacagattgacagggactttgctggtgtagtctttttataggtttt
ataaaccacttgagcctatatcagtcgttttagtgtctgacctaatatttggagctatca
gtgctttgttgatttagatgatgactcaagatttttctggtccatttcccatttccttt
tcttccctgacccccataccctcacccttaaaattctcctgtaactcaactaacaaaatc
aagcctgattcaaaacatcctagggtgttttaaacacaccatctggtgccaaatgaagat
ttttaggagtgattactaattatcaagggcacagttgtggtactgtcattgataataata
tagtttttttttttttcctaattttgacctgtttcaccagtgttttacccttgactgccc
cttctaacgacgatccaaaagtgatagtgtgtgtaagatttttaccttcctttctaaagt
ttttttttttttttttaagtgagtcctgttcttcctatttctttcagcagaaatgaaat
cccaggtaagtataagtattcaagtatttgatcagtaagtcacagttatctccagtgcat
taaataaccttcatcaagaaataggttataggtaaaatctctgaaggatcatctatgtat
tcaagtaattatttttttagataataactgtcttctggacttggtcttgaagtctgtacag
attcagcctcagtagtagcgaactgcactgctgtttggtttggagtacaaattagactta
tagtcctcctggaacttgagttattaaaatcataggaataaaattatgggatctcaacaa
agggtcgagggtttgaggcttaaacaagccaacatatgaatatatgttttgtctcgctat
actgcacttacgctatccagttgcaggtaattttttgtctgctagtagtgttctagatta
tgtctttccaaagcgctgaggctgtgcacctattctgtagttgcagctgatgcctgaatg
tatcctagctgacaaattattgattaataagaacttgaatttctggaagattcttactgt

taaccaaattttgagcaaggagtctcaaaggtaattctgaaccagaattacatgttaatg
aacagtgtaccttttaacagtgtaaatcacggaatatccgtgaagggatttcttaattta
ttttttaccggttgattgaaatatcagttaaaggttgccagcatggttgcagataaactg
atgtttgaaattcgctgaaatacttaatgtggaataggataatatacttccaatgccctc
aaggctgtgaccttacagccattttacatagcacatcattcctcctatagggatgaactt
tttcctggcacgaaaagtagccgctctggttgaagctttgcttattgtaacaggctttta
tttccaggtaatatgtcttggaagacttaattctgattagagatatagatattactggaa
actaattgttttttttctattgtactctgctttatcaaagaagtaaaacatttaaatcgt
actacagaaattaagatgttgtcttgcgatccttaataaatgaatgatttccctttaa

mutation targeting region 1220
ataaagactgcacaggagaattacccctatacctgagcctcaaccttctgggggaaaggg
aactagataacatacttcttacttgtctgtacagtaccttgttgcagatgggtgatatat
aatggtaatagaatagcacagccagacttgcttcctgcatggtagggagagacacaaaag
atgggaaactgcttttccacaaggaatctccgtagaattttgcggcgaccagatggtgca
taggtctggaaggtctgatctcccttggtcttccatgggatggttagtgtggaggggaga
tatagattgtccggccgctttgtgattccatggattgattcagtcttctggatttttttt
tctttatattttgggtactggagcttttaaaaatgtttggtttcaggtatttttattcat
gtgaagtgtatatgattctcttgagataaggttttaagctaaaatgttactccctgtttt
agtttctgaactctgacagattgacagggactttgctggtgtagtctttttataggtttt
ataaaccacttgagcctatatcagtcgttttagtgtctgacctaatatttggagctatca
gtgctttgttgatttagatgatgactcaagatttttctggtccatttcccatttccttt
tcttccctgacccccataccctcacccttaaaattctcctgtaactcaactaacaaaatc
aagcctgattcaaaacatcctagggtgttttaaacacaccatctggtgccaaatgaagat
ttttaggagtgattactaattatcaagggcacagttgtggtactgtcattgataataata
tagtttttttttttttcctaattttgacctgtttcaccagtgttttacccttgactgccc
cttctatgctgcttccaaaagtgatagtgtgtgtaagattttaccttcctttctaaagt
tttttttttttttttttaagtgagtcctgttcttcctatttctttcagcagaaatgaaat
cccaggtaagtataagtattcaagtatttgatcagtaagtcacagttatctccagtgcat
taaataaccttcatcaagaaataggttataggtaaaatctctgaaggatcatctatgtat
tcaagtaattattttttagataataactgtcttctggacttggtcttgaagtctgtacag
attcagcctcagtagtagcgtactgctcacgactatggtttggagtacaaattagactta
tagtcctcctggaacttgagttattaaaatcataggaataaaattatgggatctcaacaa
agggtcgagggtttgaggcttaaacaagccaacatatgaatatatgttttgtctcgctat
actgcacttacgctatccagttgcaggtaattttttgtctgctagtagtgttctagatta
tgtctttccaaagcgctgaggctgtgcacctattctgtagttgcagctgatgcctgaatg
tatcctagctgacaaattattgattaataagaacttgaatttctggaagattcttactgt
taaccaaattttgagcaaggagtctcaaaggtaattctgaaccagaattacatgttaatg
aacagtgtaccttttaacagtgtaaatcacggaatatccgtgaagggatttcttaattta
ttttttaccggttgattgaaatatcagttaaaggttgccagcatggttgcagataaactg
atgtttgaaattcgctgaaatacttaatgtggaataggataatatacttccaatgccctc
aaggctgtgaccttacagccattttacatagcacatcattcctcctatagggatgaactt

tttcctggcacgaaaagtagccgctctggttgaagctttgcttattgtaacaggcttta
tttccaggtaatatgtcttggaagacttaattctgattagagatatagatattactggaa
actaattgtttttttctattgtactctgctttatcaaagaagtaaaacatttaaatcgt
actacagaaattaagatgttgtcttgcgatccttaataaatgaatgatttccctttaa

Figure S1: OGT 3′UTR vector. (**A**) Vector map for the Genecopoeia vector containing the 3′UTR of OGT. (**B**) The sequence of the 3′UTR of OGT. The entire sequence of the OGT 3′UTR was inserted into the vector. Four separate vectors were utilized containing unique 3′ UTRs: wild type, mutation targeting region 513, mutation targeting region 907, and mutation targeting region 1220.

(A)

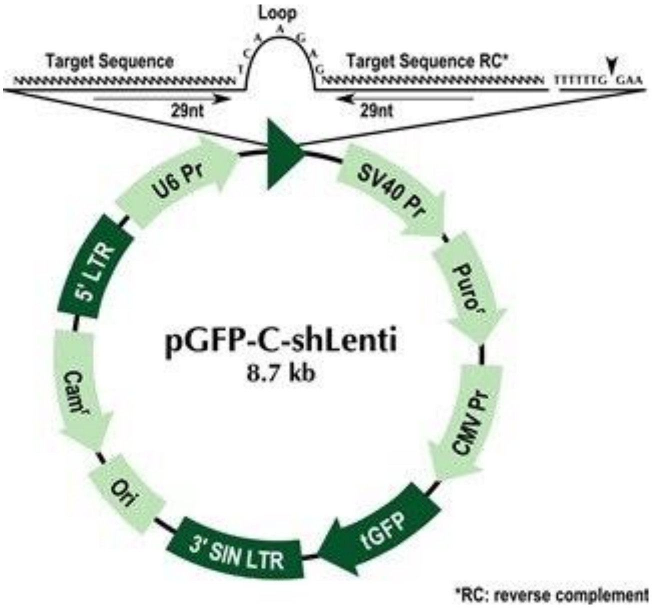

(B)

TL302811VB – CTGACCACATGATTAAGCCTGTTGAAGTC (utilized in cell line 786-O)

TL302811VD – TCCTGATGCTTATTGTAACTTGGCTCATT (utilized in cell line HTB-46)

TR30021V Lenti shRNA scramble control particles – GCACTACCAGAGCTAACTCAGATAGTACT

Figure S2: shRNA OGT vector. (**A**) Map for the Origene Lenti Cloning Vector (pGFP-C-shLenti Vector) with shRNA against OGT. (**B**) The shRNA sequences utilized in this study.

Figure 1E

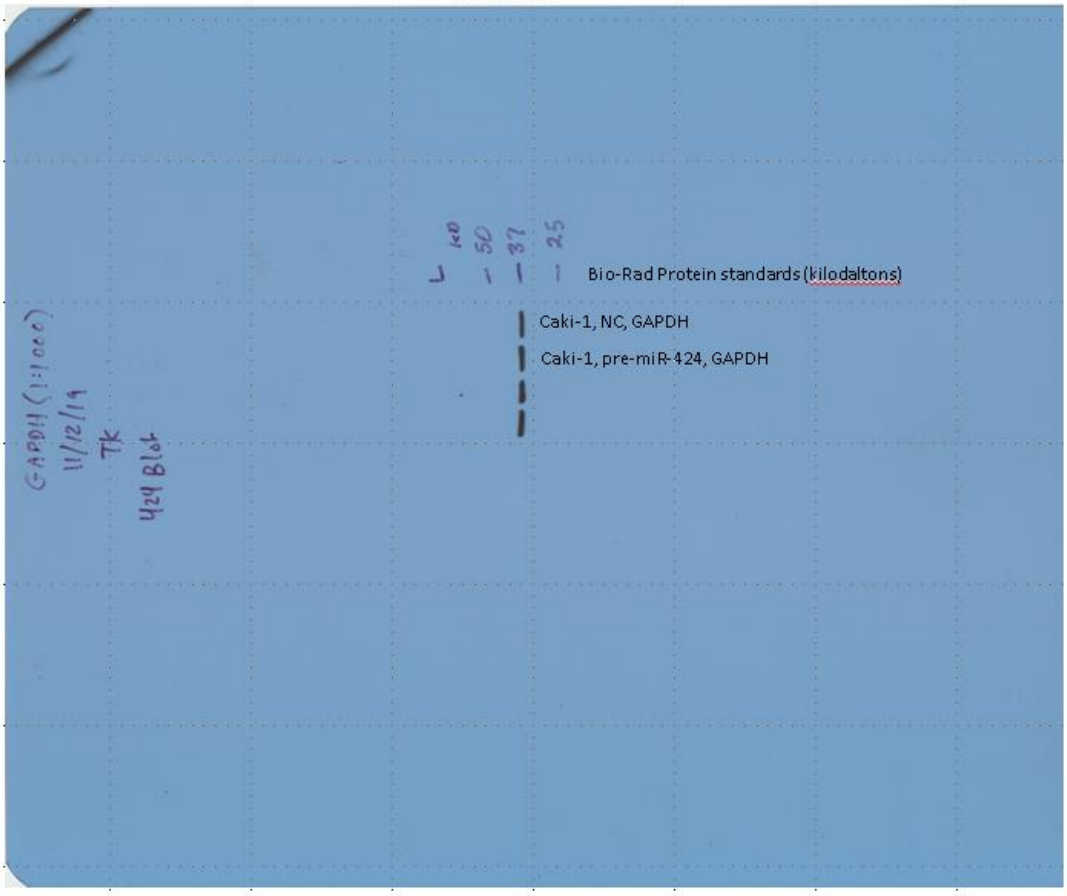

Figure S3.: Original representative blot for the GAPDH protein expression of cell line Caki-1 transfected with pre-miR-424-5p after 48 h

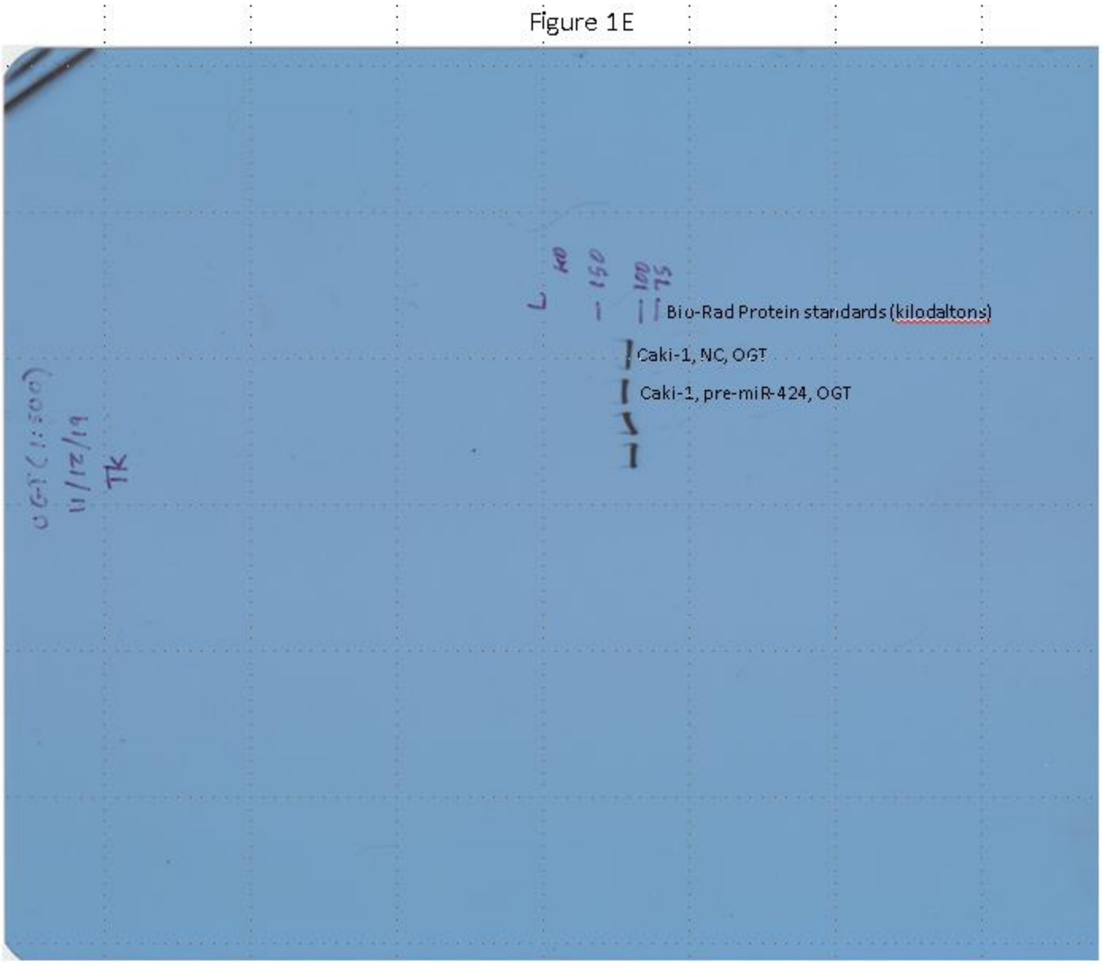

Figure S4.: Original representative blot for the OGT protein expression of cell line Caki-1 transfected with pre-miR-424-5p after 48 h

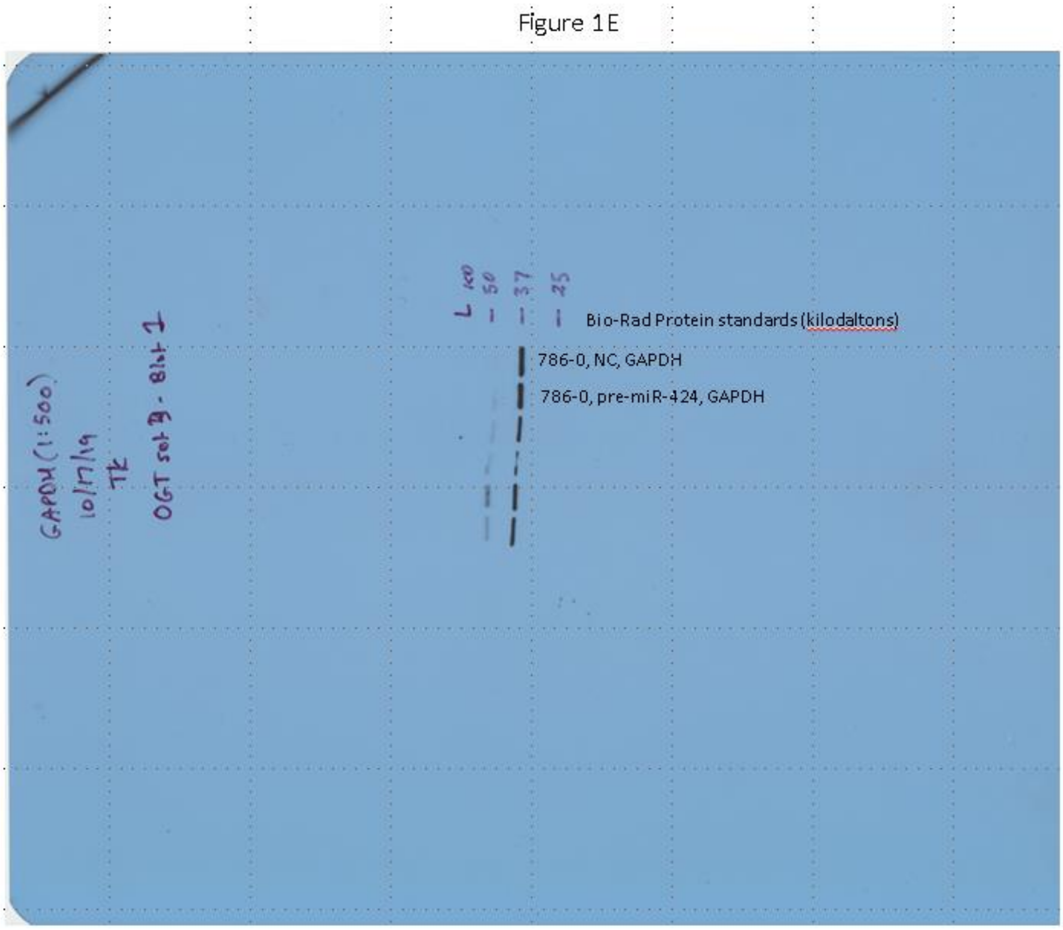

Figure S5.: Original representative blot for the GAPDH protein expression of cell line 786-O transfected with pre-miR-424-5p after 48 h

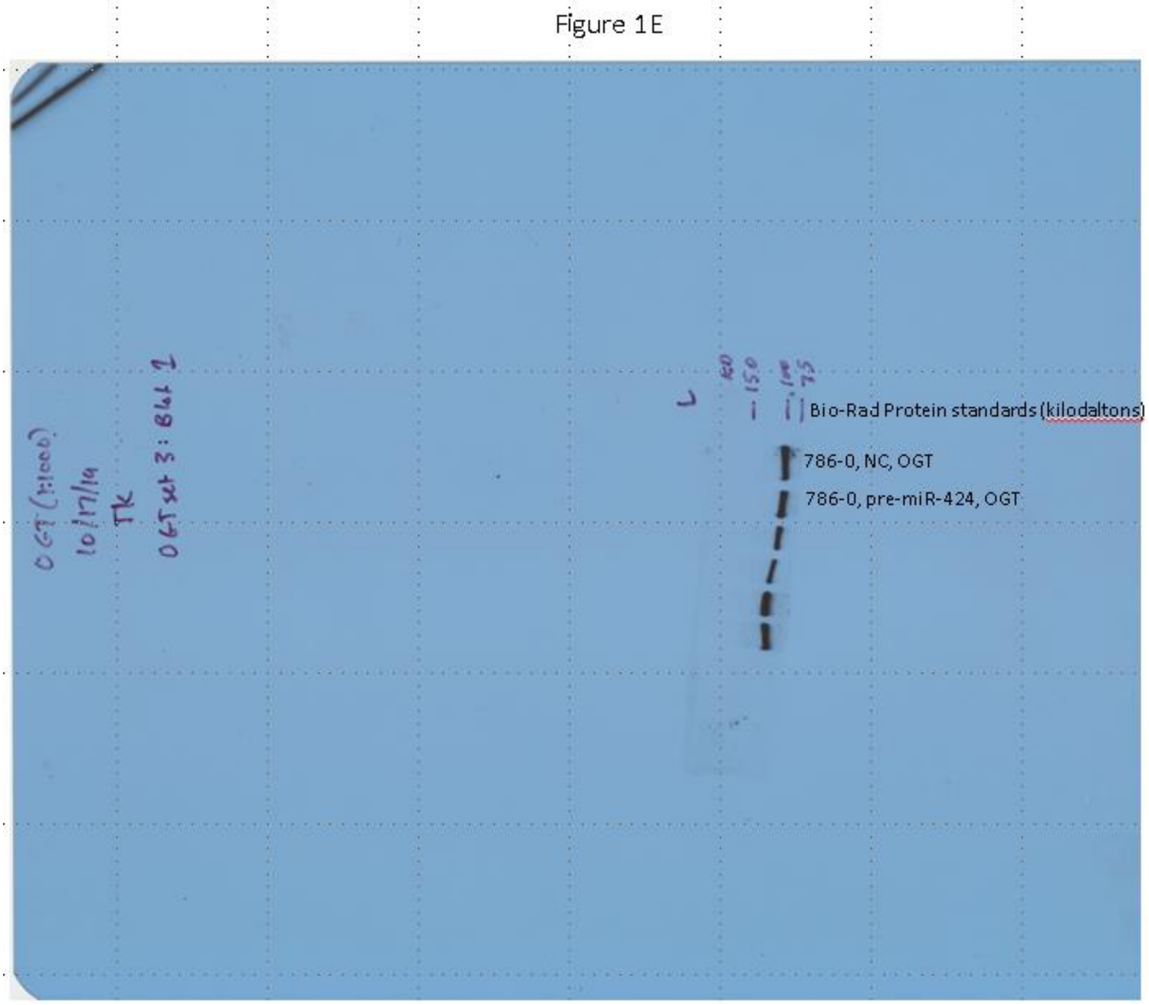

Figure S6.: Original representative blot for the OGT protein expression of cell line 786-O transfected with pre-miR-424-5p after 48

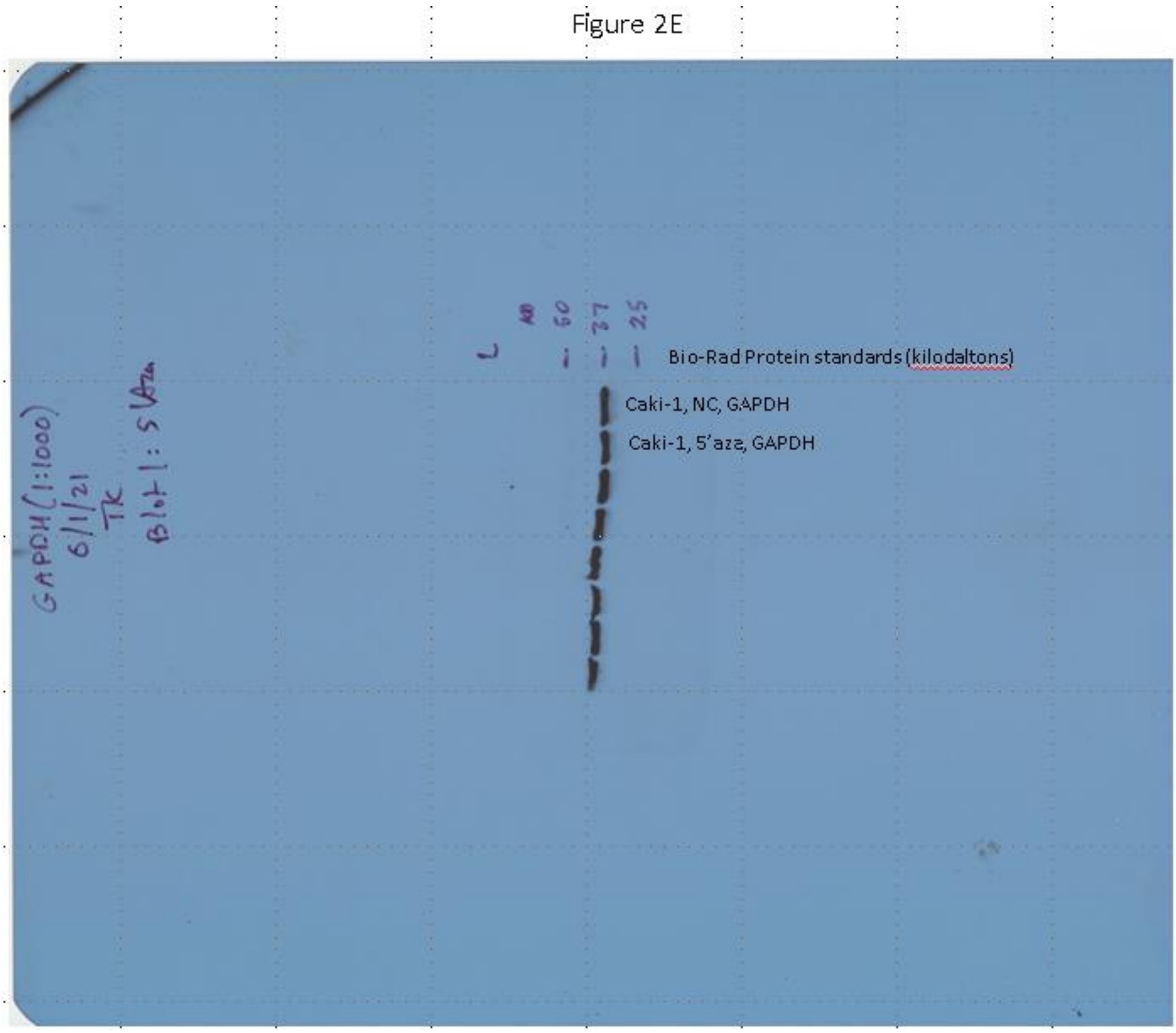

Figure S7.: Original representative blot for the GAPDH protein expression of the cell line Caki-1 treated with 5′aza after 48 h

Figure 2E

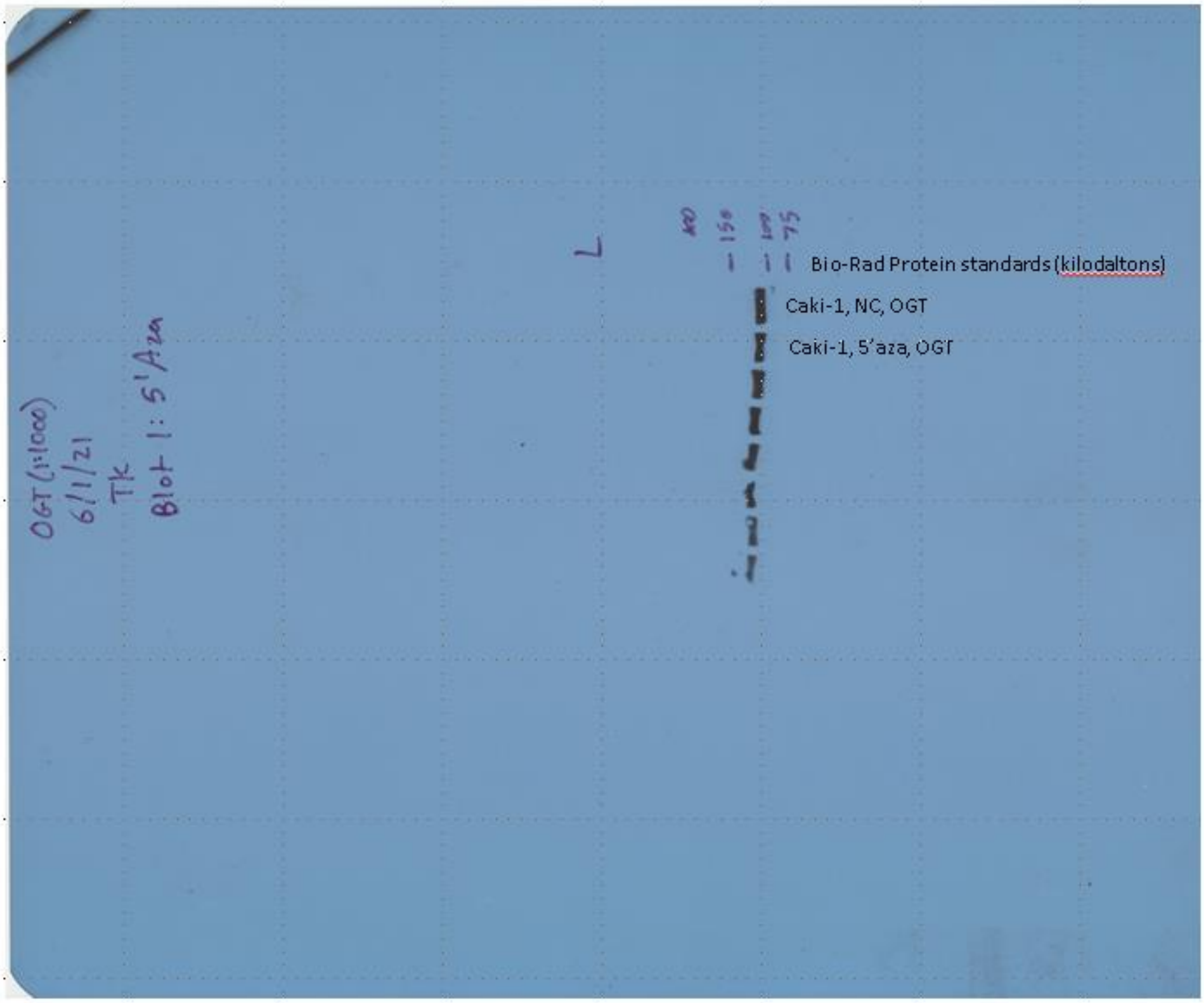

Figure S8.: Original representative blot for the OGT protein expression of the cell line Caki-1 treated with 5′aza after 48 h

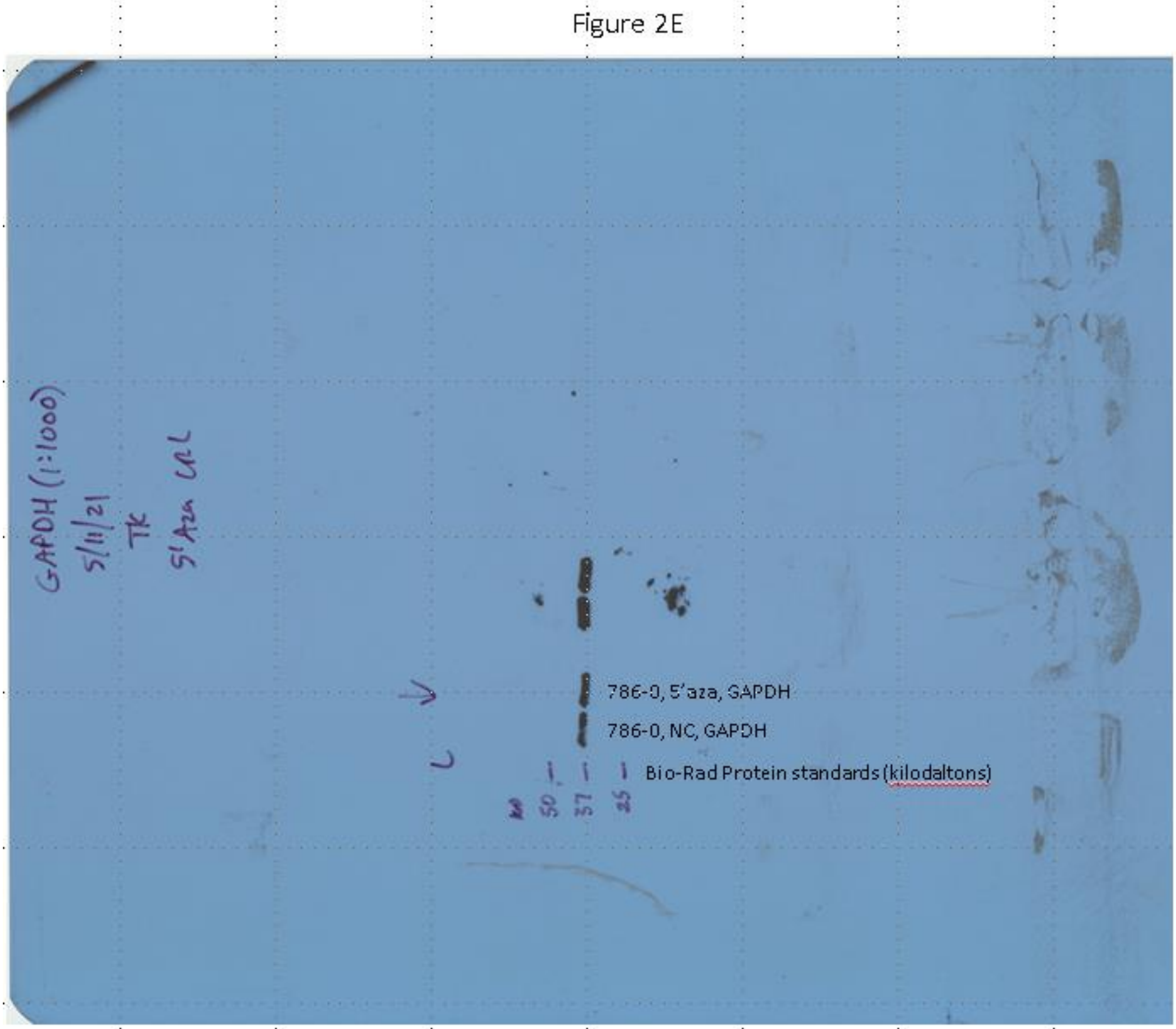

Figure S9.: Original representative blot for the GAPDH protein expression of the cell line 786-O treated with 5′aza after 48h

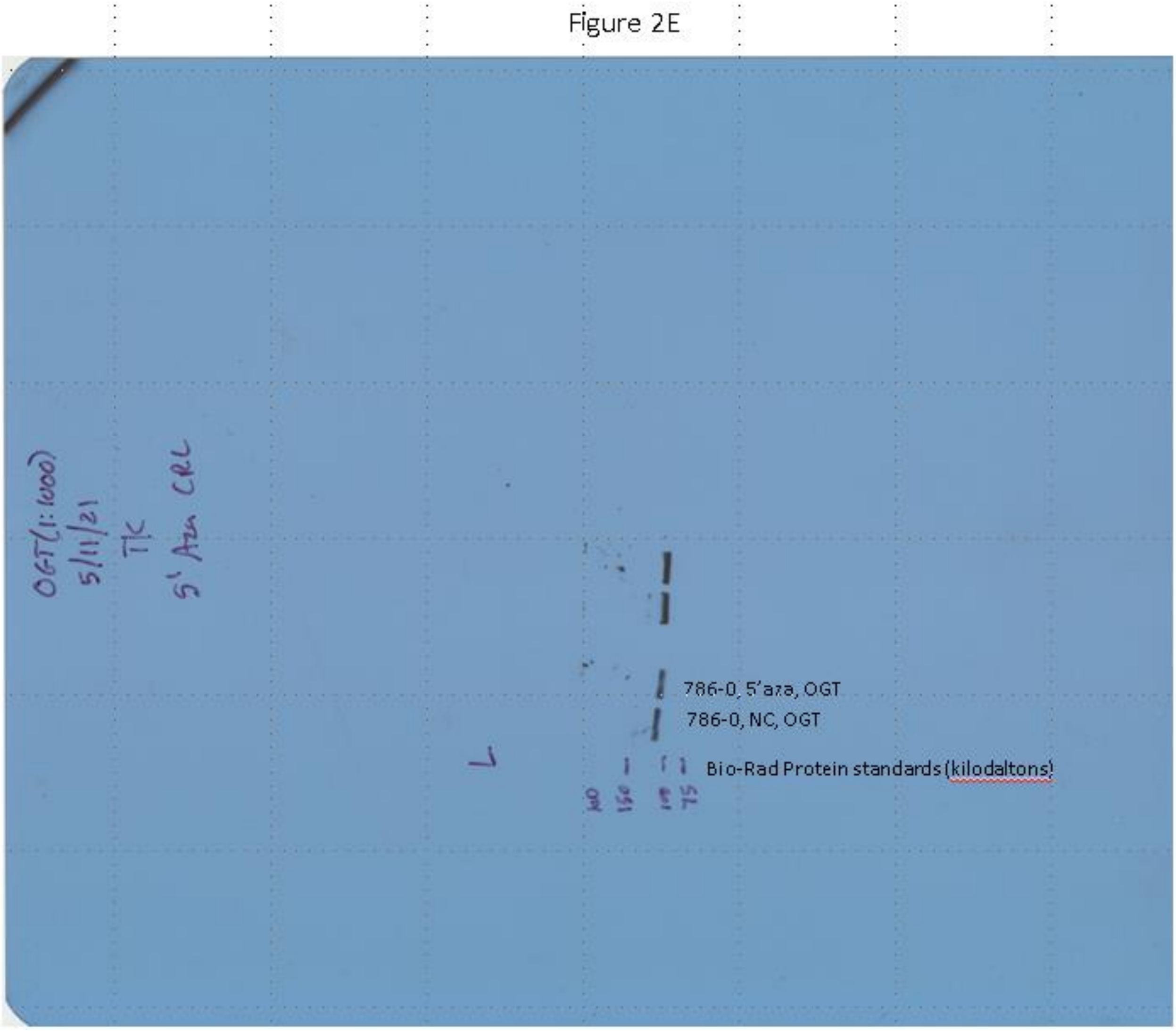

Figure S10.: Original representative blot for the OGT protein expression of the cell line 786-O treated with 5′aza after 48h

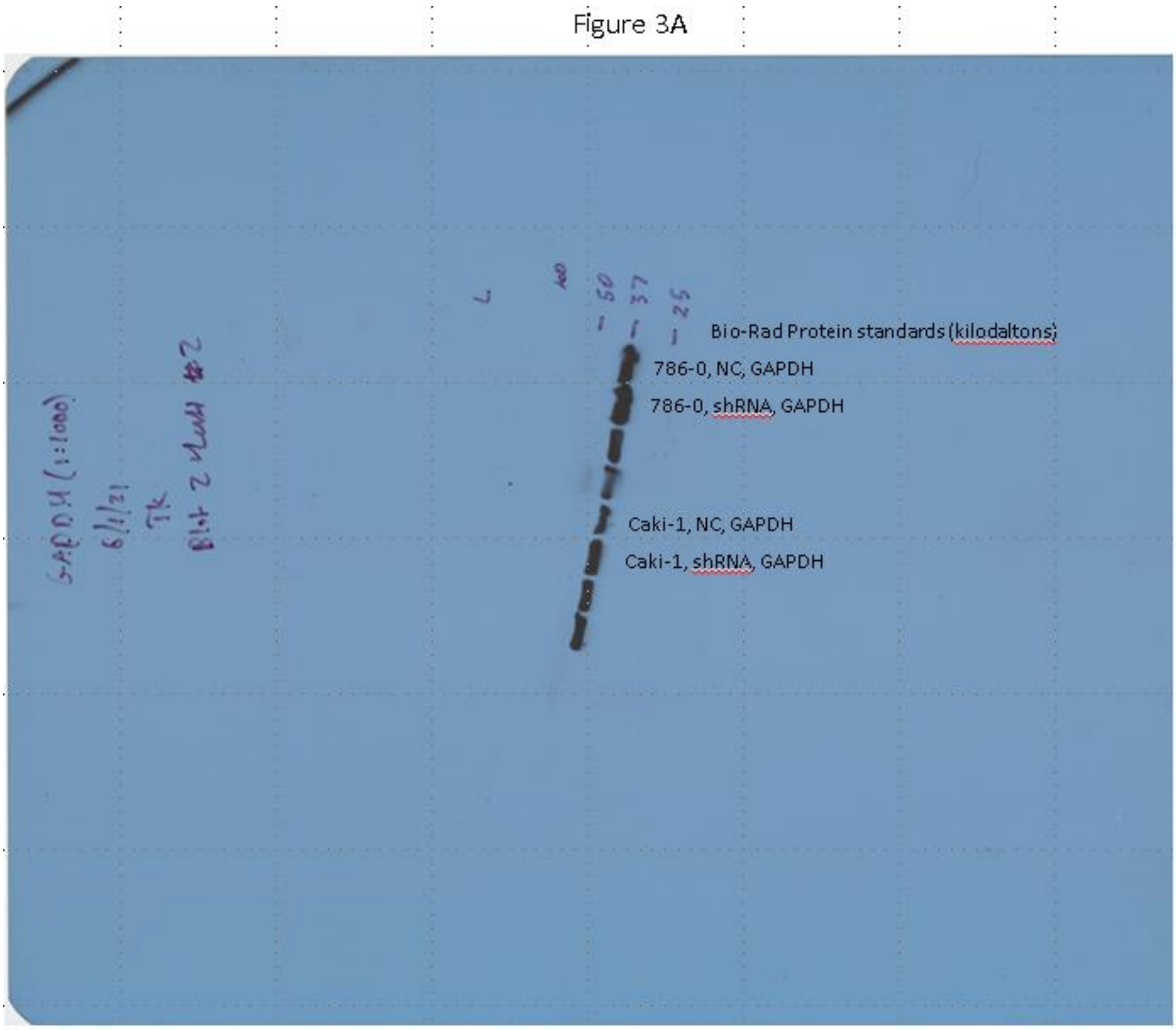

Figure S11.: Original representative blot for the GAPDH protein expression of the cell lines 786-O and Caki-1 treated with OGT shRNA

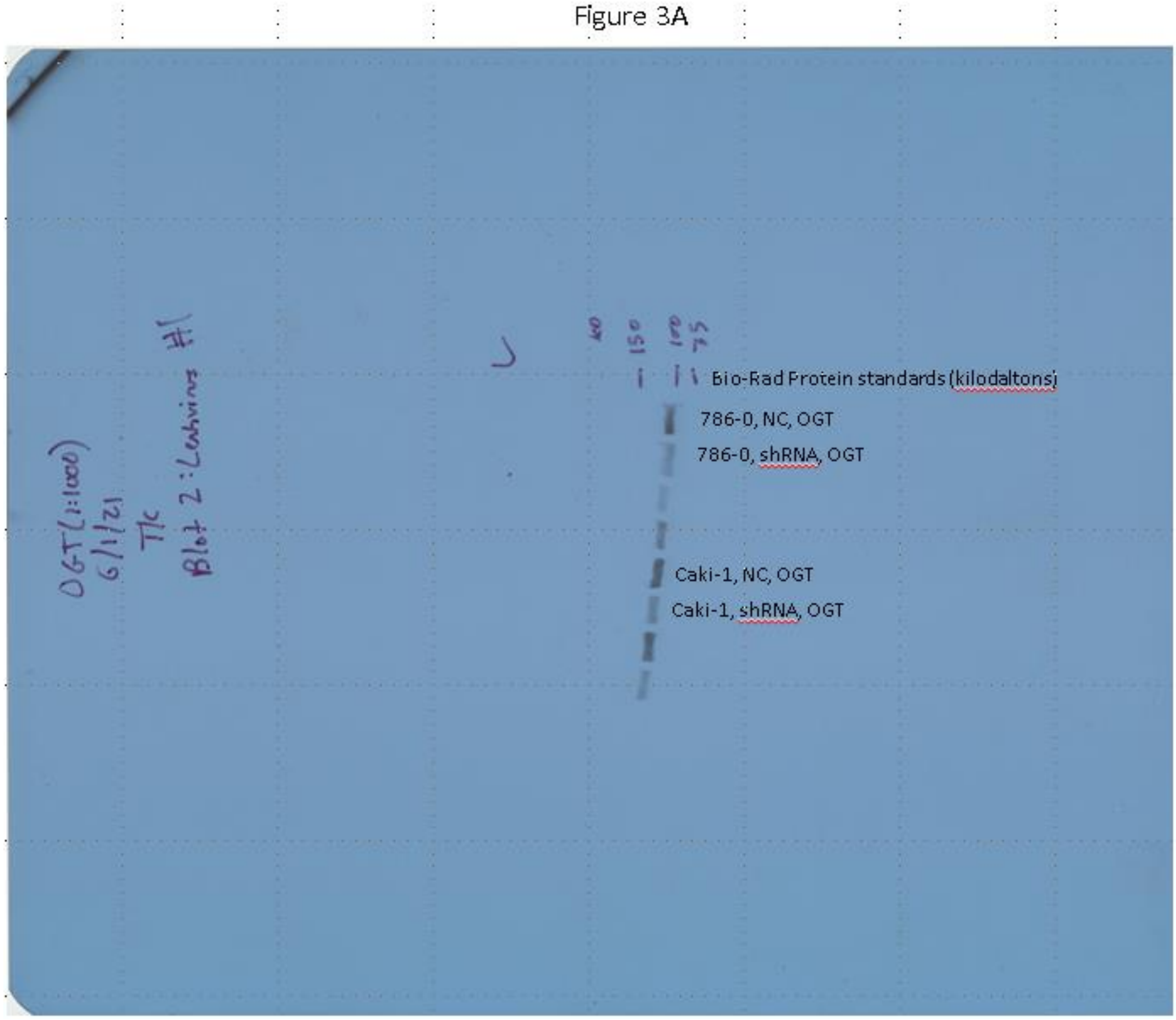

Figure S12.: Original representative blot for the OGT protein expression of the cell lines 786-O and Caki-1 treated with OGT shRNA
